# Insights on the Current State and Future Outlook of AI in Health Care: Expert Interview Study

**DOI:** 10.2196/47353

**Published:** 2023-10-31

**Authors:** Pia Hummelsberger, Timo K Koch, Sabrina Rauh, Julia Dorn, Eva Lermer, Martina Raue, Matthias F C Hudecek, Andreas Schicho, Errol Colak, Marzyeh Ghassemi, Susanne Gaube

**Affiliations:** 1 LMU Center for Leadership and People Management Department of Psychology LMU Munich Munich Germany; 2 Department of Psychology LMU Munich Munich Germany; 3 Department of Business Psychology Technical University of Applied Sciences Augsburg Augsburg Germany; 4 MIT AgeLab Massachusetts Institute of Technology Cambridge, MA United States; 5 Department of Experimental Psychology University of Regensburg Regensburg Germany; 6 Department of Radiology University Hospital Regensburg Regensburg Germany; 7 Li Ka Shing Knowledge Institute St. Michael’s Hospital Unity Health Toronto Toronto, ON Canada; 8 Department of Medical Imaging St. Michael’s Hospital Unity Health Toronto Toronto, ON Canada; 9 Department of Medical Imaging Faculty of Medicine University of Toronto Toronto, ON Canada; 10 Electrical Engineering and Computer Science Institute for Medical Engineering and Science Massachusetts Institute of Technology Cambridge, MA United States; 11 Vector Institute Toronto, ON Canada; 12 UCL Global Business School for Health University College London London United Kingdom

**Keywords:** artificial intelligence, AI, machine learning, health care, digital health technology, technology implementation, expert interviews, mixed methods, topic modeling

## Abstract

**Background:**

Artificial intelligence (AI) is often promoted as a potential solution for many challenges health care systems face worldwide. However, its implementation in clinical practice lags behind its technological development.

**Objective:**

This study aims to gain insights into the current state and prospects of AI technology from the stakeholders most directly involved in its adoption in the health care sector whose perspectives have received limited attention in research to date.

**Methods:**

For this purpose, the perspectives of AI researchers and health care IT professionals in North America and Western Europe were collected and compared for profession-specific and regional differences. In this preregistered, mixed methods, cross-sectional study, 23 experts were interviewed using a semistructured guide. Data from the interviews were analyzed using deductive and inductive qualitative methods for the thematic analysis along with topic modeling to identify latent topics.

**Results:**

Through our thematic analysis, four major categories emerged: (1) the current state of AI systems in health care, (2) the criteria and requirements for implementing AI systems in health care, (3) the challenges in implementing AI systems in health care, and (4) the prospects of the technology. Experts discussed the capabilities and limitations of current AI systems in health care in addition to their prevalence and regional differences. Several criteria and requirements deemed necessary for the successful implementation of AI systems were identified, including the technology’s performance and security, smooth system integration and human-AI interaction, costs, stakeholder involvement, and employee training. However, regulatory, logistical, and technical issues were identified as the most critical barriers to an effective technology implementation process. In the future, our experts predicted both various threats and many opportunities related to AI technology in the health care sector.

**Conclusions:**

Our work provides new insights into the current state, criteria, challenges, and outlook for implementing AI technology in health care from the perspective of AI researchers and IT professionals in North America and Western Europe. For the full potential of AI-enabled technologies to be exploited and for them to contribute to solving current health care challenges, critical implementation criteria must be met, and all groups involved in the process must work together.

## Introduction

### Background

Rising life expectancy, increasing prevalence of noncommunicable diseases (eg, diabetes), and staffing shortages are among the most severe challenges health care systems face worldwide [1]. As a result, the demand for health care services is steadily increasing, and health care costs are soaring [2,3]. Moreover, the high demand for services, extensive administrative and documentation requirements, and staffing shortages lead to heavy workloads for health care workers (HCWs) and reduce the time that staff can spend with patients and performing actual medical duties [4]. These circumstances jeopardize patient safety and limit the overall ability to deliver health care services [5-8].

The use of health technologies has often been suggested as a possible solution to address these challenges. By improving workflows, relieving staff of routine tasks, and reducing the frequency of medication errors and medical errors in general [8], health technologies might help ensure better health outcomes and increase efficiency [9,10]. In particular, artificial intelligence (AI) through machine learning has increasingly become the focus of health IT development in recent years. Health care professionals and patients associate AI technology with improved care [11,12] and reduced workloads [11,13,14]. Numerous high-performing AI algorithms have been developed to support HCWs with various tasks in different medical fields, such as radiology, cardiology, neurology, ophthalmology, oncology, gastroenterology, mental health, and many others [15-17].

However, despite the extensive research on AI applications in health care, the implementation of AI-enabled clinical decision support systems (AI-CDSSs) in clinical practice lags behind what would be feasible according to technical developments [18]. Several explanations for the slow adoption of AI systems in health care have already been proposed. Various groups are involved in this AI implementation process: (1) policy makers and authorities who determine the framework conditions for the entire process; (2) researchers and developers who develop, train, and market the system with its various functions; and (3) IT experts in health care facilities who sometimes make decisions about system acquisitions, integrate them into the existing infrastructure and maintain them if necessary, and introduce them to the (4) HCWs who ultimately use the system in their everyday work [19,20]. Many issues have been brought forward by or are attributed to 2 groups of people on both ends of the technology implementation spectrum: HCWs and policy makers, both of whom are essential for the success of AI technologies in health care.

On one side of the spectrum, physicians and other HCWs are the end users of most AI systems in health care. The technology is developed to support their workflows, but if HCWs are reluctant to use AI systems, the proposed advantages of the technology cannot materialize [21,22]. On the one hand, HCWs believe that AI has the potential to improve the quality of care through more accurate and precise diagnoses, as well as enabling faster diagnoses and shorter wait times. It can also promote personalized care tailored to the patient and ensure greater consistency in diagnoses as the performance of AI technologies does not suffer from human stress symptoms, fatigue, or difficulty concentrating [23,24]. HCWs also expect collaboration with AI-enabled systems to reduce daily workload and save staff time by allowing the technology to prioritize symptoms and patients and provide legal protection for medical staff through ongoing documentation of the care process [23,24]. On the other hand, however, research has shown that current and future HCWs are reluctant to use AI applications in their daily work for a variety of reasons. These include concerns about the performance of the technology and fears that overtechnologization may impair their abilities over time as AI takes over tasks and clinicians become overly reliant and accustomed to the technology [13,23]. Some HCWs also suspect that AI systems will influence staff diagnostic decisions [23] and fear that technology will make their jobs redundant [25,26]. In addition, HCWs are concerned that using these technologies will negatively affect the physician-patient relationship and might compromise privacy as AI systems would have to work with patients’ sensitive data [23].

On the other side of the spectrum are policy makers (eg, intragovernmental and governmental organizations as well as regulatory bodies such as the US Food and Drug Administration [FDA] or the European Medicines Agency). They are responsible for the ethical, legal, and regulatory frameworks and conditions for the implementation of AI systems in health care. Policy-making bodies have already issued guidelines on AI implementation and have discussed unresolved legal and regulatory issues such as certification [27], liability, and data protection [28]. Moreover, policy makers have expressed concern about ethical issues such as discrimination and lack of transparency, which might hinder the safe and widespread implementation of AI applications in health care [27].

However, when it comes to the physical implementation of AI technology into the existing health care infrastructure, in most cases, neither policy makers nor HCWs are directly involved in the process. In reality, the 2 other stakeholder groups (ie, researchers and developers as well as IT experts) are responsible for the practical implementation of AI products in health care facilities. Researchers have discussed many challenges surrounding AI systems in health care. Some of these are naturally linked to the issues raised by the other stakeholder groups, such as the lack of trust among users [21,29]; regulatory burdens; and concerns about accountability, ethical data use, biases, and discrimination [30]. Other mentioned challenges relate to more technical issues such as unsatisfactory system performance, detection of biased data, system explainability, cost and quality of labeled data, and computational limitations [30,31]. The perspective of IT professionals has received considerably less attention in the literature. Some research has shown that they see the lack of human, professional, and financial resources and incompatibility with existing IT infrastructures as barriers to implementing AI technologies in health care [32]. In addition to an acceptable user interface and robust connectivity to the infrastructure, AI researchers and developers are searching for contact with clinical users [33].

Besides looking at the various stakeholder groups, it is important to consider regional differences when trying to obtain a global perspective on the current state of AI implementation in health care. International comparisons show substantial differences in overall investment in developing and deploying new AI technologies. Overall, the United States and China have raised the most venture capital funds, followed by Europe, which, however, lags significantly behind the former 2 [34]. When looking specifically at health care–related investment in AI technology, again, the United States, China, and Europe are the 3 global players, which is also reflected by their amount of research output [35]. One study has already conducted a cross-regional comparison of the adoption of AI in small- and medium-sized health enterprises between Germany and China. It showed that Germany-based professionals named challenges related to data accessibility, transparency, and regulations more often than their Chinese colleagues [36]. To the best of our knowledge, no study has systematically compared European and North American experts’ views on the opportunities and challenges of implementing AI applications in health care.

### Objectives

This study focused only on the 2 professional groups closest to the physical integration of AI systems. We wanted to mention that the topic should ideally be viewed more holistically. According to the Responsible Innovation and Responsible Research and Innovation approaches, it is important to involve all stakeholders to prioritize the ethical, social, and sustainable aspects of technological advances. This is to ensure that innovation and research benefit society while minimizing harm and accounting for societal needs and values [37].

By exploring the implementation of AI technologies in health care, we wanted to focus on stakeholders directly involved in the process. Researchers’ perspectives have been discussed extensively in the literature but have mainly focused on potential opportunities, technical challenges, and ethical issues of AI models rather than their implementation. In contrast, the views of IT professionals in health care have received little attention overall. To fill this gap, this study used a mixed methods approach to collect and compare the opinions of researchers and IT professionals on implementing AI technology in health care from their respective points of view. In addition, we included respondents from North America and Europe to uncover potential regional differences in addition to profession-specific differences.

## Methods

### Sample

The 2 critical inclusion criteria for participating in this preregistered study were *profession* and *region*. We focused on researchers working on AI applications for health care and medicine and IT professionals in the health care sector. These professional groups allowed us to obtain the views and differences in opinions of 2 key stakeholders directly involved in the implementation of AI applications in health care practice. The researcher group consisted of computer scientists and clinical scientists ranging from senior doctoral candidates to faculty members. The group of IT experts included chief technical officers and chief information officers from hospitals, representatives of medical device safety organizations, and chief executive officers of health IT companies. The 2 regions of interest were Western Europe and North America, with the European countries of Germany, Austria, Switzerland, and Belgium and the North American countries of the United States and Canada being represented. By including participants from these Western regions, we were able to gain valuable insights into different legal and health care systems and highlight regional differences between these global players. As the 2 professional groups are highly specific, no other selection criteria or prerequisites, such as minimum professional experience, were stipulated. Ultimately, 23 individuals were interviewed, including 13 (57%) researchers (n=7, 54% from Western Europe and n=6, 46% from North America) and 10 (43%) IT experts (n=8, 80% from Western Europe and n=2, 20% from North America).

### Recruitment

Sampling was performed via a web search based on relevant publications and matching of LinkedIn profiles. In addition, experts were recruited via snowball sampling through the authors’ networks and recommendations from participants and other third parties. The participants were selected on a nonprobabilistic basis, that is, deliberately according to the aforementioned criteria [38]. We planned to interview at least 20 experts, balanced between professional groups and regions, to obtain a well-rounded picture of the topic. A total of 104 candidates were approached via email, of whom 23 (22% participation rate) agreed to participate. Interviewees received no compensation for their participation.

### Data Collection

Data for this cross-sectional, mixed methods study were collected using semistructured expert interviews. Consequently, all participants received the same questions from the interview guide, with the option of the interviewers asking follow-up questions or using prompts if needed. The authors developed the interview guide for this study based on the research questions and the literature presented in the *Introduction* section. It included questions from four categories: (1) the prevalence of AI applications in hospitals and the current state of the technology, (2) their implementation criteria, (3) the challenges, and (4) the potential of implementing AI systems in health care. The original interview guide was pretested twice, resulting in minor improvements. Between November 2021 and January 2022, all 23 interviews were conducted remotely via Zoom (version 5.8.3-5.9.1; Zoom Video Communications, Inc) and by phone. The interviews lasted between 14.5 and 49.5 (mean 30.0, SD 8.0) minutes and were conducted in English (19/23, 83%) and, at the request of the interviewees, in German (4/23, 17%). At the start, participants were informed about the purpose, procedure, expected duration, voluntary nature of the interview, and how their data would be processed. The interviewees provided informed consent to participate in the study and for the interviews to be recorded. At the beginning of the recording, the participants were first asked to briefly describe their professional backgrounds as an icebreaker. This was followed by our predefined interview questions and, if needed, follow-up questions and prompts. After discussing all the questions, participants had the opportunity to add anything they felt was relevant to the topic. At the end of the interview, we asked for other potential interviewees and thanked the interviewees for their participation.

### Data Preparation

Every participant received a nonidentifiable acronym under which their materials were stored and analyzed. The acronym only indicated the person’s professional group and region, which was needed for the analysis (researcher in North America [RENA], researcher in Western Europe [REEU], IT expert in North America [ITNA], and IT expert in Western Europe [ITEU]). The interview recordings were transcribed using Trint (version unknown; Trint Limited). Contextual information that could lead to the identification of an individual was manually anonymized in the transcripts. All transcripts were reviewed, translated into English if necessary, and uploaded to MAXQDA (version 20.4.2; VERBI Software GmbH). The raw material with sensitive data, that is, consent forms and audio or video files, was securely stored in a password-protected digital folder.

### Data Analysis

For thematic analysis of the data, we used MAXQDA. We chose a combination of deductive and inductive qualitative methods [39]. This approach integrates a theory-driven template [40] and a data-driven framework [41] for developing codes. The method includes 6 steps for data analysis, the details of which can be found in the literature [39] and in an extra document in the study’s repository on the Open Science Framework [42]. At the end of the thematic analysis process, 14 cross-cutting themes and 172 subthemes were identified, divided into 4 categories, and captured in the final codebook. Three additional themes were identified in the *challenges* category: interdisciplinary work, ethics, and user. However, these were much smaller in scope than the other themes and, therefore, were not considered further in the rest of the study. To validate the coding process, a third previously uninvolved author analyzed a representative subsample of 10% (10/100) of the data using the final coding manual [43,44]. The intercoder agreement with a code overlap in segments of at least 90% (90/100) was a Cohen κ value of 0.77, which is considered a substantial match [45,46]. After the second coding, only minor changes were made to the final codebook.

Following the qualitative thematic analysis, we also analyzed the interviewees’ responses quantitatively using topic modeling to identify latent topics as well as the most frequently used words. Quantitative text analysis has been found to be a useful tool for validating results of previous qualitative analysis [47-49]. In this case, we first removed the introductory and closing parts of the interviews that only contained introductions and small talk. Furthermore, we deleted all stop words, which are words that are commonly used with little or no relevance to the content of a text (eg, “and” and “did”). We also singularized all words (ie, “algorithms” became “algorithm”). Then, we computed the frequencies of words (uni-, bi-, and trigrams) grouped by interviewees’ region and profession. For a better visual illustration, these were plotted in word clouds. On the basis of the findings from the qualitative analyses that had been validated by quantitative analysis, we extracted 14 topics using latent Dirichlet allocation [50] with Gibbs sampling (Cronbach α=.30) [51]. Finally, we manually matched the qualitative themes with the quantitatively extracted topics with regard to their content. All text data processing and statistical analyses were performed using the statistical software R (version 4.1.1; R Foundation for Statistical Computing). Specifically, we used the R packages *udpipe* for tokenization [52] and *topicmodels* as well as *ldatuning* for topic modeling [49,53].

The following documents can be found in the Open Science Framework repository [42]: the preregistration, the list of participants, the interview instructions and the interview guide in English and German, the description of the qualitative coding process, the final codebook, a table showing the frequency of themes and subthemes, and a table of the top 10 words identified during quantitative topic modeling.

### Ethical Considerations

This study was exempt from a full ethical review by Committee on the Use of Humans as Experimental Subjects, the institutional review board of the Massachusetts Institute of Technology, by meeting the criteria for exemption (E-4248).

## Results

### Overview

Four broad categories emerged during the qualitative analysis: (1) the current state of AI technology in health care, (2) the implementation criteria and requirements of AI systems in health care, (3) the challenges in implementing AI technology in health care, and (4) the technology’s outlook. Table 1 provides an overview of the most relevant aspects that emerged from the qualitative (themes) and quantitative (topics) analyses clustered within these 4 categories. Table 2 provides a more detailed overview of the 14 topics that emerged from the quantitative analysis, each with the top 5 underlying words. Initially, we present a quantitative overview of the interview content of each expert group. This is followed by an in-depth look at the most relevant qualitative themes. Subthemes that fall under several themes are described only once.

The 14 topics extracted through the quantitative analysis of our interview data matched well with many themes from the qualitative analysis: prevalence (topics 1 and 7), regional differences (topic 2), capabilities (topics 3 and 7), limitations (topic 5), performance and safety (topics 5 and 9), system integration and human-AI interaction (topics 8 and 12), costs (topic 11), stakeholder involvement (topic 11), employee training (topic 4), different kinds of challenges (topics 3, 11, 12, and 13), threats (topic 11), and opportunities (topics 6, 10, and 14).

**Table 1:** Relevant themes and topics from the qualitative and quantitative analyses.

Category and theme (qualitative)		Topic (quantitative)
**Current state of AI^a^ systems in health care**		
	Prevalence	AI in health care AI in medical imaging^b^
	Regional differences	Regional challenges^b^
	Capabilities	Improving the everyday experience of HCWs^c^ AI in medical imaging^b^
	Limitations	Clinical research^b^
**Implementation criteria and requirements of AI systems in health care**		
	Performance and safety	Clinical research^b^ Performance
	System integration and human-AI interaction	Workflow optimization Human-AI interaction^b^
	Costs	Barriers to AI implementation^b^
	Stakeholder involvement	Barriers to AI implementation^b^
	Employee training	Employee training
**Challenges in implementing AI systems in health care**		
	Regulatory challenges	Regional challenges^b^
	Logistical challenges	Barriers to AI implementation^b^
	Technical challenges	Human-AI interaction^b^ Industry challenges
**Outlook**		
	Threats	Barriers to AI implementation^b^
	Opportunities	Future developments Technical advances Opportunities

^a^AI: artificial intelligence.

^b^These quantitative topics can be assigned to several qualitative themes.

^c^HCW: health care worker.

**Table 2:** A total of 14 quantitatively extracted topics from the interview transcripts.

Topic name	Topic description	5 most frequent words^a^
AI^b^ in health care	Impact of AI adoption on health outcomes	health care, perspective, health, challenge, learn
Regional challenges	Regulatory challenges for AI implementation in certain regions	germany, company, clinic, regulatory, country
Improving the everyday experience of HCWs^c^	Integrating AI into routine patient care for the benefit of HCWs	patient, time, nurse, care, day
Employee training	Training HCWs on how the system works and its limitations	system, decision, implement, training, medical
Clinical research	Studying the risks and benefits of AI in clinical settings	algorithm, physician, clinician, study, risk
Future developments	Exploring long-term solutions and regulatory aspects for diagnostic development	solution, term, future stuff, united states
AI in medical imaging	Current AI applications in radiology and medical imaging	image, radiologist, radiology, diagnosis, application
Workflow optimization	Improving institutional workflows and communications with AI	model, question, understand, talking, sense
Performance	Impact of AI on performance and practices	human, data, performance, practice, super
Technical advances	Using technology to facilitate knowledge-based change in medicine	technology, field simply, machine learning, change
Barriers to AI implementation	Logistical and stakeholder challenges in implementing AI	person, situation cost, university, feel
Human-AI interaction	User-centered technology integration to support HCWs	hospital, doctor, environment, person, issue
Industry challenges	Industry challenges in deploying AI systems	process, level, wrong, set, improve
Opportunities	Creating opportunities for data-driven clinical care in specific domains	data, clinical, basically, care, answer

^a^The 10 most frequent words are included in the project repository.

^b^AI: artificial intelligence.

^c^HCW: health care worker.

### Quantitative Overview of Regions and Professions

Table 3 shows the 10 most frequently used words divided by professional (researchers and IT experts) and regional groups (Western Europe and North America). The higher the words are ranked in the table, the more frequently they were mentioned by the interviewed experts.

**Table 3:** Ten most frequently used words grouped by profession and region of the interviewees.

Profession	Western Europe	North America
Researchers	person data patient system algorithm doctor germany hospital process human	algorithm person data patient hospital healthcare human time care model
IT experts	patient algorithm data hospital company person germany question time diagnosis	technology clinical health adoption clinic level model opportunity augmentation challenge

### Current State of AI Systems in Health Care

#### Prevalence of AI Systems in Use

Respondents named 12 different medical fields or specialties in which AI algorithms have been developed for clinical practice: neurology, oncology, radiology, dermatology, cytomorphology, surgery, pediatrics, pathology, ophthalmology, urology, genomics, and diabetology, as well as intensive care medicine. Many interviewees focused on AI systems for radiology, which could indicate that this is the most mature field for the technology. Classification of medical imaging was often mentioned as a relevant use case, again potentially highlighting the maturity of this application. Interviewees also mentioned discipline-independent use cases. For instance, current AI systems can also use text-based data from electronic health records (EHRs) to make medical predictions using natural language processing. AI algorithms are also used to optimize administrative tasks such as staff scheduling and billing. However, 48% (11/23) of the interviewees acknowledged that many systems are not commercially available but are at the stage of in-house scientific research projects. Almost exclusively, European interviewees emphasized that systems are not widely used in routine clinical practice:

We see projects on the scientific side where we use AI. But I couldn’t describe a single use case where a real AI, some kind of neural network deep learning mechanism, would be in place in our normal health care activities.

ITEU18; position 7

#### Regional Differences in Research and Development

The interviewees mentioned the United States and China as leaders in AI research, whereas Germany and many European countries seemed to lag behind. Within Europe, the Nordic and Baltic countries, as well as the United Kingdom, are considered frontrunners in AI development for the health care sector:

So if you look at places like Singapore and also China, you will also see that this area of [sic] analyzing huge amounts of data and applying algorithms from AI [sic] to novel case [sic], this is something where they are, I would say, even years ahead of what we [Germany] are doing.

ITEU18; position 11

Several reasons were given for why European AI research is trailing that of the United States and China. Researchers and IT experts primarily blamed the lack of available data for training the models caused by stricter data protection laws and regulations. The General Data Protection Regulation (GDPR) implemented in the European Union in 2018 makes sharing data between research and health care facilities within and across countries more complicated:

I think that GDPR...makes it a little more difficult for data sharing in Europe. And so that may be part of why the research is not...progressing quite as fast.

RENA05; position 20

Researchers from Europe further pointed to the slow progress of digitalization in health care and the lack of financial investments as barriers to the advancement of AI-enabled systems:

Germany is lagging behind due to digitalization...a switch from spreadsheets to platforms that really integrate patient data is really needed in Germany.

REEU10; position 13

#### Capabilities of Current AI Systems

Both professional groups referred to similar technical capabilities. Currently, AI algorithms can support HCWs mostly in 2 ways. First, the systems can perform specific, highly repetitive tasks that are easy but time-consuming for humans. Consequently, deploying these applications can reduce workload and free up time for other tasks:

I should say here...that the AI applications are usually very narrow based, which means that they can do a simple task...But it’s automated, so it might go faster, which is easier for the radiologist.

ITEU08; position 48

Second, AI systems outperform humans when working with large and complex data. This type of data is often characterized by a diffuse structure, complex interrelationships, and multidimensionality. By instantly incorporating more data than a human ever could, AI algorithms can make faster and more accurate predictions:

The way these algorithms work is they can handle...complexity that we as humans can’t.

RENA16; position 33

#### Limitations of Current AI Systems

Both researchers and IT professionals described the fact that AI algorithms currently cannot operate without human supervision as a major limitation. At the moment, it is required that HCWs verify the algorithms’ results. Thus, the full responsibility and liability for clinical decision-making remain with the user:

The limits, obviously, are [sic] they can’t take responsibility for what they’re doing...They can’t take any responsibility in terms of medical legal issues. So whenever you do something...some poor doctor has to sign the whole thing and then he’s responsible for whatever happens.

REEU07; position 28

Another limiting factor mentioned by both groups was the technology’s high task specificity, which limits its usefulness in 2 ways. On the one hand, AI algorithms are often programmed to use only one source of information (eg, 1 type of medical image) for their prediction, whereas integrating multiple sources (eg, medical images and patient history) would yield better results. On the other hand, medical decisions often require the involvement of multiple disciplines, for example, radiology and surgery. Consequently, integrating several stand-alone algorithms or developing multitask algorithms would be needed to support the entire workflow for multidisciplinary teams:

That’s just that...the AI system is trained for a specific use case, for example skin cancer, then it looks at the skin image, but does not include other things, from the case history or similar large.

ITEU09; position 24

One fundamental limitation mentioned by IT professionals was the lack of explainability of currently deployed AI algorithms. The absence of information on how the algorithm operates makes it difficult for users to understand why a specific recommendation or prediction was made, which might make them skeptical of relying on it:

So it’s always the case that they say the systems are great, but mostly they can’t explain them reasonably. That means that one of the current limits is the ability to explain how the decision was actually made.

ITEU09; position 23

### Implementation Criteria and Requirements for AI Systems in Health Care

#### Performance and Safety

Both professional groups mentioned high performance in the form of a low error rate most frequently as the primary criterion for adopting AI-enabled systems. Accordingly, algorithms should only be implemented in health care settings if they show high accuracy to ensure patient safety:

Because human lives are at stake here. Currently, there is simply no time for trivialities, but it must work 100.0%. And that’s why over 99.0, so 99.5/99.8 are the requirements for implementing the AI system.

ITEU03; position 28

Some interviewees advocated comparing the performance of algorithms with human performance and evaluating them using the same standards. However, in reality, users seem to have much higher performance expectations of AI systems than of humans. Therefore, the experts argued that algorithms with a performance that matches or exceeds that of human experts, even if they are not always perfect, might help improve overall decision accuracy:

If I have an algorithm whose AUC is .94. Really [sic] good performing algorithm. But the clinicians perform better. Their AUC is .96. It’s not a good algorithm because you’re not outperforming clinicians. But if you’ve got an algorithm where the performance isn’t very good, their AUC is .68. But the clinician AUC is .58. It’s a good algorithm because it does better than the clinicians.

RENA16; position 38

Researchers emphasized that algorithms must be revalidated when deployed in a new environment as local data might differ from the training data. Therefore, all stakeholders involved in the implementation process must ensure that the algorithms perform well in new environments and over time:

Ideally, you would do a revalidation of that algorithm on your institution’s data, your patient population.

RENA12; position 24

#### System Integration and Human-AI Interaction

The experts pointed out that the successful deployment of AI systems depends on how easily they can be integrated into the existing technical infrastructure of the respective institution:

And how easy is it to integrate within the system? How much time does it take to do that? How many and how can we see the results? How can it be integrated in our reports, for example?

ITEU08; position 64

Both professional groups considered good usability and smooth workflow integration to be as important as performance for using AI technology in health care. According to the experts, users will only be willing to engage with a new technology if it makes their work easier. System interfaces should be designed intuitively enough for users to operate without substantial training and must be adaptable to users’ needs:

For the nurses, we actually had to develop an interface that they wanted to see. That’s very simple for them to work with. So keeping things as simple as possible.

RENA16; position 48

According to 35% (8/23) of the respondents, users’ acceptance of and trust in the technology is another essential factor (or barrier if missing) before purchasing and deploying AI systems in health care settings. Without the end users’ acceptance and willingness to use these systems, the implementation process is doomed to fail:

But [work] culture is way more important [than performance]. So culture first, do they actually want to use this stuff? Are they open-minded and they want to embrace that?

RENA16; position 37

Several researchers even suggested that AI technology should work only in the background, automatically taking signals from all the different data streams, integrating them, and acting accordingly without human intervention. This would make the system much easier to use and bypass complex human-technology interaction issues. The interviewees claimed that background operationality might be particularly advantageous regarding user acceptance as issues of trust in the technology might not even arise in solely background operating systems. In addition, less effort and fewer resources are needed to introduce the AI system to users if they are not directly interacting with it:

So when you look at what the future would hold, what’s actually going to get adopted, I think they’re going to be solutions that are doing operational things where the healthcare workers are not interacting in a deliberate way with those systems.

RENA15; position 29

Although many experts argued that AI must be explainable so that users know how and why the system makes a prediction, some IT professionals completely disagreed. According to them, staff do not even need to know whether the underlying technology is AI-enabled so that they handle all devices unbiased:

Nobody should know there’s an AI model inside. Is [sic] not relevant.

ITNA04; position 33

#### Costs

Costs were also mentioned as a criterion by both professional groups. However, the interviewees disagreed on how important this factor is:

I would say, this is not the major factor. I mean, costs are always a factor, but in the end, it has to be evidence-based. In the end, you have to understand what is the outcome of using such an algorithm.

ITEU18; position 21

I’m going to have to cough up a lot of money and then when am I going to see the value of this? So it’s really important when it comes to the implementation that there is a very clear business case and value proposition of why this matters now, both near-term and long term.

ITNA02; position 26

#### Stakeholder Involvement

There was only partial consensus on which actors are the most important for the implementation of AI systems in health care. This could be because technology procurement processes vary widely across health care facilities. Differences were also found between professional groups as well as between regions.

In both regions, the institution or department heads appear to be the driving force behind technology adoption. European researchers assumed that finance departments also play a role in purchasing decisions:

So in the end it’s always the heads of the institutes or the chief physicians who have to say yes...So I would say that they are the ones who mainly have to be convinced.

ITEU09; position 32

Researchers from North America stated that hospitals’ IT professionals are involved in the implementation:

The other stakeholders are typically the people who manage the...computer systems and the people who would have to set it up and install it.

RENA12; position 31

Interviewees from both professions and regions indicated that regulatory bodies are important stakeholders for implementing AI in health care:

If it’s not built in the institution, you would have to go to actual regulatory approval. Certainly, if this system is going to have a direct impact on patient care.

ITNA04; position 22

Moreover, some respondents also mentioned that the actual end users, meaning patients, might be a relevant stakeholder group for successfully implementing AI systems in health care:

And there again, we have the question: are we allowed to do so? Is it something the patient has to agree for and so on? So these are all criteria to choose.

ITEU18; position 21

#### Employee Training

Nearly all experts emphasized the need for basic AI skills and knowledge so that users can safely interact with the systems and recognize their limitations. It has been argued that training on AI should be integrated into the curricula for current and future HCWs who will work with AI-enhanced systems:

Part of the education of our workforce, will include the basics of how these systems work, where they fail, where they can potentially cause harm.

RENA15; position 31

However, participants disagreed on how much training is needed. Some thought that HCWs need to be able to operate the AI systems and understand their underlying mechanisms, including functions and limitations. Consequently, training should start as early as possible, preferably already during the education period. Other experts thought that training should be limited to the most necessary information to minimize the burden on staff. In particular, the level of training should be adapted to the complexity of the AI system and the learning culture within institutions:

So what we are trying to do is to have students, first of all, be aware of artificial intelligence and what it is, what it can do, what it can’t. Then different techniques like, for example, what is computer vision? How does that work? So what is object recognition? Then further on with natural language processing.

REEU10; position 48

### Challenges in Implementing AI Systems in Health Care

#### Regulatory Challenges

Data protection and security emerged as the primary regulatory challenges. Strict regulations limit access to data needed to develop advanced algorithms. Interviewees from Europe especially lamented that the inability to share data across institutions hinders AI research and implementation:

When you take machine learning...the regulatory challenges are the data protection regulation.

ITEU22; position 35

Moreover, the experts mentioned that certification processes, especially FDA approval for medical products, are a significant challenge for developers. Documentation guidelines interfere with the continuous improvement of the algorithm once systems have been deployed:

Does he have the certificate? Has the constancy test been carried out? Every small deviation in patient monitoring must be documented, and this is also queried, sometimes half a year later, although the patient has long left.

ITEU03; position 47

Predominantly, IT professionals were concerned about liability issues in cases when the system fails and incorrect decisions are made as a consequence:

And you can ask the question who’s liable: the hospital or is [sic] the company that created the model? And we haven’t seen the first lawsuit yet.

ITNA04; position 48

Although regulations can slow down the development and implementation of AI systems in health care, some experts said that they are necessary to ensure patient safety:

Well, the regulatory process is inherently conservative...as slow as it needs to be to make sure that we stay safe and that’s appropriate.

RENA05; position 48

#### Logistical Challenges

Securing funding for AI algorithms was the most frequently cited logistical challenge. Both developers and medical institutions face high costs in developing, acquiring, implementing, and maintaining new systems:

The costs of healthcare are rising and rising in Germany and in other countries, too, so hospitals do not have all the money in the world to introduce the systems.

REEU06; position 45

I know that the implementation is going rather slow, and for the vendors, it’s slower than expected, which also makes it quite difficult for them because they have to invest a lot of money, and they have invested. But they also would like to see a return on investment, of course.

ITEU08; position 33

The lack of IT professionals needed to implement AI-enabled systems into existing IT infrastructure was also mentioned as a huge barrier. In addition, in-house data scientists who can monitor and operate the systems are required, placing even greater human resources and financial burdens on institutions:

And this may include lack of access to IT resources and personnel, right, skilled people.

RENA15; position 18

Some researchers pointed out that health care institutions need to collaborate more to improve AI algorithms and unlock their real potential. Collaboration mainly involves sharing and integrating data across institutions as, at the moment, important data for optimal predictions are lost for an algorithm when patients change institutions during their treatment. However, sharing and integrating sensitive data is particularly complex and resource intensive:

Healthcare is not a single point event. It’s a process. And so somebody will go to his doctor and will get a potential diagnosis. We get some diagnostic workup. We’ll go to the specialist, we’ll get some more diagnostic work up. The information from the primary doctor gets lost.

REEU07; position 62

#### Technical Challenges

Researchers identified the lack of available high-quality preprocessed training data and data on rare diseases as a major challenge affecting the algorithm’s performance. In addition, using unprocessed hospital data (eg, data coming directly from EHRs), which would be more readily available, is challenging as these data are not standardized:

So you have label data and unlabeled data, and the labeled data is usually labeled by human experts. And the quality of the model always depends on whether or not the labels are accurate.

REEU10; position 18

IT professionals were concerned about biases in the training data that could distort the algorithms and make their predictions less accurate for people who were underrepresented in the training data. Biases in the form of under- or overrepresentation of certain patient and disease groups can occur. For instance, wealthy and renowned hospitals, which are regularly involved in generating training data, have a nonrepresentative patient and disease pool. This is especially problematic as it is challenging to detect biases in the data in the first place and to correct the model at the operational level:

It’s also very difficult to identify whether there is a certain bias involved. If you have a large set of data and we know that there are typically some biases and there is research to identify biases, but there’s very often a hidden bias which you cannot automatically detect.

ITEU18; position 18

Researchers also complained about the poor and inflexible IT infrastructure that makes the implementation of AI algorithms challenging:

And then when we speak about technical challenges, it’s more about the hardware, to be honest, because although this is not always available in medical institutions.

REEU20; position 32

Moreover, some interviewees mentioned that AI developers struggle to design AI system interfaces that meet user needs in the complex health care environment. Currently, the systems often fail to provide user-centered and user-friendly designs:

Then the other challenge is designing the human interaction in the [sic] way that people can actually use it.

REEU20; position 33

### Outlook

#### Threats

Researchers in particular expressed great concern about the possibility that the deployment of AI-enabled systems might exacerbate health care disparities that already exist in society. There are several reasons for this. As mentioned previously, biases in the algorithm’s training data might lead to less accurate algorithmic predictions for underrepresented, often marginalized groups, which might cause serious harm. Moreover, health care facilities in wealthier regions tend to be the first to adopt new technologies. As a result, their patients will benefit from AI innovations, whereas patients in poorer areas will be left further behind:

There’s a substantial risk for creating new or exacerbating existing racial, sexual and socioeconomic healthcare disparities.

RENA12; position 56

Another threat mentioned only by researchers was automation bias, which is the tendency to rely too much on AI-CDSSs. As a result, system users may fail to detect prediction errors if they accept AI advice unconditionally. Consequently, automation bias poses a danger if the algorithm is not highly reliable, which could lead to many medical errors:

You can also have things go the other way where people put, you know, way too much trust in the AI, and they kind of, you know, blindly...trust whatever it’s saying. Even...if they’d stopped and thought about it, they would realize that the result that was coming out is nonsensical.

RENA12; position 55

IT professionals expressed concerns about cyberattacks as AI systems in health care are also not immune to hacking. Cyberattacks could affect both data security and patient outcomes if the algorithms are compromised or unavailable because of the attack:

We had some hacker attacks in the history, in the last 5 years in some hospitals in Europe and if systems are not available, then still all the work flows need to be working. And if you rely too much on AI and digitalization, of course, it’s a problem.

REEU06; position 48

Although it is often discussed that AI systems could make some jobs obsolete, our interviewees unanimously predicted that the adoption of AI technology will not lead to job losses in health care in the near future. However, task-specific skills that require a lot of training might decline if AI systems are widely used:

So whereas in the early days in the media, you could read AI will replace radiologists. Well, this is of course not true because looking at a CT scan of the lungs is much more than only counting nodules.

ITEU08; position 48

If you have a system that supports you a lot, you may also have the risk to lose [sic] your own skill in a situation of doubt that can be very harmful.

REEU20; position 24

#### Opportunities

According to the experts, the most significant opportunity for using AI systems in health care is the reduction in workload. For instance, outsourcing time-consuming and repetitive tasks to an AI system would allow HCWs to focus on more complex tasks and patient interactions:

When it’s implemented in a very good way and the doctors have trust, it frees time for direct communication with the patient.

ITEU22; position 48

IT experts saw tremendous opportunities in AI technology to improve diagnostic accuracy and patient outcomes through decision support. In addition, AI algorithms could enable truly personalized health care by analyzing multiple sources of health data simultaneously and across time. For instance, long-term EHRs could be combined with vital signs recorded via digital devices and analyzed using an algorithm. Long-term integrated data analysis could potentially facilitate the early detection of previously hidden disease patterns and provide individualized prevention and treatment plans:

So I do think that AI will be able to provide a more specific and more patient-specific treatment based upon the information, the data that we obtain.

ITEU08; position 75

Researchers pointed out that health care logistics such as supply chain management and billing could benefit from AI systems. AI algorithms are already used in other industries to support logistical, administrative, and planning processes:

There’s a lot of opportunity for AI in supply chain, billing, claims management.

ITNA04; position 44

## Discussion

### Principal Findings and Comparison With Prior Work

Plenty of research on the challenges and opportunities of AI technology in health care has been published. However, our novel approach of pooling the expertise of AI researchers and IT professionals from Western Europe and North America resulted in a novel, nuanced, and comprehensive overview based on four main categories: (1) the current state, (2) implementation criteria and requirements, (3) implementation challenges, and (4) future outlook.

Within the current state theme, the interviewees mentioned that AI systems have been developed for various medical fields and use cases, primarily image classification in radiology and pathology, but have yet to be widely deployed in clinical practice. According to the literature, the 3 global players in health AI are the United States, China, and Europe, with the former 2 investing the most in research and development [34-36]. Our experts agreed that the United States and China dominate research and development but emphasized much more that Europe lags behind, largely because of lower investment in technology and digitalization and limited access to data because of stricter privacy regulations. At the moment, AI systems can support clinical decisions and diagnoses by providing predictions for specific tasks. Previous studies have found that many HCWs believe that AI systems will improve diagnostic accuracy as the technology does not have classic human limitations such as fatigue and difficulty concentrating [23,24]. Our experts agreed that the use of AI technology can improve diagnostic accuracy but stated that the main reason for this improvement is the fact that AI systems are better at dealing with large and complex data than humans. In addition, although some HCWs expressed hope that relying on AI systems might provide legal protections [23], our experts explained that AI-CDSSs currently cannot operate without human oversight, are sometimes inaccurate, and lack both explainability and accountability. Consequently, liability fully remains with the HCWs operating the system, a current limitation of the technology widely discussed in the literature [30,54,55].

From the interviews, several critical implementation criteria and requirements emerged. In accordance with the literature [10,30], the interviewed experts agreed that high performance is the essential criterion for implementing AI-enabled systems in health care. Ideally, deployed algorithms should outperform human experts, explain their predictions, be approved by regulatory bodies, and be frequently revalidated. Easy and unintrusive integration into existing infrastructures and workflows, intuitive and user-friendly design, and high user acceptance were frequently mentioned as essential requirements. Specifically, lack of trust and user acceptance have also been widely discussed in the existing literature as major problems for the successful adoption of AI technology in health care [21,29]. There was consensus among our interviewees that the involvement of health care facility leaders, regulatory bodies, and end users is critical to AI adoption. Moreover, experts emphasized that users require training to interact safely with the technology. By already integrating the topic of AI in health care into the medical curriculum, users can develop the knowledge and understanding, especially of the limitations, and the confidence needed to use AI in a clinical setting [56].

The interviewees identified multiple challenges in implementing AI systems in health care. Many mentioned strict data protection and security regulations, complex certification processes, and the unresolved question of liability as fundamental regulatory challenges to technological development and deployment. These regulatory aspects have been discussed in previous research, especially from the side of policy makers. In addition, previous work has focused on ethical considerations such as the lack of transparency and discrimination in the context of AI-CDSSs in health care [21,29]. The experts agreed on several significant logistical challenges such as procuring funding for AI systems, the lack of capable IT professionals needed for technology implementation and maintenance, and difficulties with sharing and integrating data across institutions. From a technical standpoint, the lack of available preprocessed, representative, high-quality data impairs the training of high-performance AI algorithms for the entire patient population. Researchers surveyed in previous studies have confirmed these challenges and also stated that useful data are expensive and often come with computational limitations [30,31]. Our interviewees mentioned that institutions’ outdated and inflexible IT infrastructures are also a big challenge for deploying AI technology. Correspondingly, IT experts in previous studies have emphasized the compatibility problems of the systems with the existing IT infrastructure [32].

The interviewed experts mentioned that implementing AI technology holds both threats and opportunities for the future. Concerns were expressed that biased training data might exacerbate health care disparities, hurting marginalized groups, and that automation bias might lead to medical errors. Moreover, AI systems in health care could become a target for cyberattacks. Previous research has shown that HCWs are concerned about losing skills and potentially even their jobs owing to AI technology. HCWs also worry about the adverse effects of using AI systems for the physician-patient relationship and patient privacy [13,23,25,26]. Our experts also acknowledged the problem of losing training-intensive skills but disagreed with the notion that AI systems will make some HCWs obsolete in the foreseeable future. In addition, they did not mention HCW-patient relationships or patient privacy as major limitations of AI systems. Overall, our experts agreed consistently with the previously mentioned opportunities that the technology could offer [8-14,23,24]: workload reduction for HCWs, improvements in diagnostic accuracy and patients’ health outcomes, and advances in personalized medicine and optimized health care logistics.

Generally, the statements of both professional groups closely coincided; however, we also found some interesting differences. IT professionals emphasized China’s leading role in AI technology more strongly than researchers. In particular, the researchers blamed the state of digitization and numerous regulations for why Europe is lagging behind. Researchers emphasized the need for high security of the system and its regular validation. Some researchers recommended simply letting AI technology work in the background. However, if not, the system should integrate smoothly into the existing workflow. Consequently, researchers called for users to know how AI systems work to understand their limitations. The 2 groups also highlighted different implementation challenges—for example, IT experts considered biased training data as one of the biggest challenges. Researchers naturally focused much more on technical challenges such as data availability, technical infrastructure, and interfaces. Interestingly, only researchers mentioned overreliance on the system as a real threat from AI technology. Finally, considering future opportunities, IT experts highlighted themes such as increasing health care service availability and improving clinical outcomes, whereas researchers focused more on reducing HCWs’ workloads.

After proportionally adjusting for the imbalance between respondents from Western Europe and North America, we found that their views differed on some topics. North American experts spoke more frequently and in more detail about the overarching themes of AI, machine learning, algorithms, and technology. Many European respondents felt that the lack of available and shareable data is the reason that AI development and adoption in Europe are slow. They exclusively indicated that lack of accountability and open liability issues were major limiting factors for using AI systems. Accordingly, answering these questions was a necessary criterion for implementing the technology. Interviewees from North America emphasized regular system validation and seamless workflow integration, ideally working only in the background, as critical implementation criteria. They also saw biased training data as one of the biggest threats to AI integration. Overall, North American respondents were more likely to talk about implementation challenges.

### Implications for Research and Practice

In total, 5 aspects emerged from the interviews that seem to be particularly important in the context of AI implementation in health care. First, data protection is a central element for AI development and adoption as it regulates access to training data and has implications for the performance of AI support tools. The problem of a lack of available and shareable data is specifically prominent in European countries. If Europe wants to keep up with the global players in AI-enabled technology for health care, a fundamental change in the rules on how data are made available, shared, and integrated across institutions will be needed. Second, all stakeholders seemed to agree that high performance is the most fundamental aspect of successfully implementing AI systems in health care. To ensure high performance in the real world, AI systems have to be continuously monitored and revalidated in the environment in which they operate. Third, as the end users of many AI systems for health care, HCWs play an important role in the successful implementation of the technology and should be prepared accordingly. HCWs should be trained on how to interact effectively and safely with the technology and learn about its limitations to avoid relying on incorrect advice. Research should be conducted to identify the most appropriate and effective strategy to train HCWs on the technology. Fourth, it is also striking that ethical concerns are hardly addressed except for data protection and possible biases within the data. Further development of AI systems in health care should necessarily take place within a defined ethical framework for action as the technologies are in direct contact with sensitive patient data and humans themselves. Finally, given that researchers and IT professionals often raise different issues on similar topics, it is important to ensure that all stakeholders involved in AI implementation collaborate and consider each other’s opinions. AI systems should be developed to meet the needs of and support practitioners in their everyday work; consequently, their views should matter the most.

### Limitations

This study has several limitations. First, the regional backgrounds of our interview partners were not perfectly balanced. Overall, more participants worked in Western Europe than in North America, with a much larger proportion of the IT experts interviewed coming from Western Europe. This might have skewed the results toward a more European-centered view. Even the proportional adjustment of the statements of the underrepresented group of experts cannot guarantee a balanced picture. Second, experts from several but not all Western European countries, let alone all European countries, were interviewed. In particular, experts from Baltic and Scandinavian countries would be of interest to the study as these regions were frequently mentioned by the interviewees as European pioneers in AI technology. In addition, the North American expert group consisted only of people who worked in the United States or Canada. Third, the focus of interviewees in the field of radiology may have been due to selection bias as several interviewees (7/23, 30%) had strong domain expertise in radiology, which is understandable as this is the field where AI technologies are commonly used. However, some aspects relevant to implementing AI-enabled systems in other medical fields may have been overlooked. Finally, inherent features of qualitative expert interview studies (including small and, to a degree, self-selected samples and nonstandardized data analysis) cannot ensure the generalizability of the results. Subsequent studies should provide a more balanced and broader field of experts and use more quantitative methods to improve generalizability. To gain an even more global view of the current state of AI systems in health care, experts from other countries, especially China and wider parts of Europe and North America, should be included in future research.

### Conclusions

Our study provides new insights into the implementation process of AI technology in health care from the perspective of AI researchers and IT professionals in North America and Western Europe. Our cross-professional and international approach revealed nuanced views on various topics from 2 stakeholder groups actively involved in the technology’s deployment. Although interviewees from both groups and regions had relatively consistent views, they often focused on different aspects that they deemed most relevant. This highlights the importance of systematically documenting technology adoption expectations and challenges from different perspectives to avoid overlooking some critical elements. Our findings provide a broad overview of the current state, criteria, challenges, and prospects for the deployment of AI technology in health care. To advance the technology and make it widely available, critical implementation criteria have to be met, and all stakeholders must collaborate to overcome the challenges hindering the technology from reaching its full potential. By designing the development processes based on participatory design principles, AI-enabled applications can truly help solve current and future problems faced by health care systems worldwide.

## Abbreviations

AI: artificial intelligence
AI-CDSS: artificial intelligence–enabled clinical decision support system
EHR: electronic health record
FDA: Food and Drug Administration
GDPR: General Data Protection Regulation
HCW: health care worker
ITEU: IT expert in Western Europe
ITNA: IT expert in North America
REEU: researcher in Western Europe
RENA: researcher in North America
